# Apolipoprotein ɛ4 Is Associated With Increased Risk of Fall- and Fracture-Related Hospitalization: The Perth Longitudinal Study of Ageing Women

**DOI:** 10.1093/gerona/glae134

**Published:** 2024-05-20

**Authors:** Jedd Pratt, Jack Dalla Via, Craig Sale, Abadi K Gebre, Blossom C M Stephan, Simon Laws, Kun Zhu, Wai H Lim, Richard L Prince, Joshua R Lewis, Marc Sim

**Affiliations:** Department of Sport and Exercise Sciences, Manchester Metropolitan University Institute of Sport, Manchester, UK; Nutrition and Health Innovation Research Institute, School of Medical and Health Sciences, Edith Cowan University, Perth, Western Australia, Australia; Department of Sport and Exercise Sciences, Manchester Metropolitan University Institute of Sport, Manchester, UK; Nutrition and Health Innovation Research Institute, School of Medical and Health Sciences, Edith Cowan University, Perth, Western Australia, Australia; School of Pharmacy, College of Health Sciences, Mekelle University, Mekelle, Tigray, Ethiopia; Institute of Mental Health, The University of Nottingham Medical School, Nottingham, UK; Dementia Centre of Excellence, enAble Institute, Curtin University, Perth, Western Australia, Australia; Centre for Precision Health, School of Medical and Health Sciences, Edith Cowan University, Joondalup, Western Australia, Australia; Collaborative Genomics and Translation Group, School of Medical and Health Sciences, Edith Cowan University, Joondalup, Western Australia, Australia; Medical School, The University of Western Australia, Perth, Western Australia, Australia; Department of Endocrinology and Diabetes, Sir Charles Gairdner Hospital, Perth, Western Australia, Australia; Nutrition and Health Innovation Research Institute, School of Medical and Health Sciences, Edith Cowan University, Perth, Western Australia, Australia; Department of Renal Medicine, Sir Charles Gairdner Hospital, Perth, Western Australia, Australia; Nutrition and Health Innovation Research Institute, School of Medical and Health Sciences, Edith Cowan University, Perth, Western Australia, Australia; Medical School, The University of Western Australia, Perth, Western Australia, Australia; Nutrition and Health Innovation Research Institute, School of Medical and Health Sciences, Edith Cowan University, Perth, Western Australia, Australia; Medical School, The University of Western Australia, Perth, Western Australia, Australia; Nutrition and Health Innovation Research Institute, School of Medical and Health Sciences, Edith Cowan University, Perth, Western Australia, Australia; Medical School, The University of Western Australia, Perth, Western Australia, Australia; (Biological Sciences Section)

**Keywords:** Women’s health, Community-dwelling, Injurious falls, Musculoskeletal

## Abstract

Apolipoprotein ɛ4 (*APOE ɛ4*) may be a genetic risk factor for reduced bone mineral density (BMD) and muscle function, which could have implications for fall and fracture risk. We examined the association between *APOE ɛ4* status and long-term fall- and fracture-related hospitalization risk in older women. A total of 1 276 community-dwelling women from the Perth Longitudinal Study of Aging Women (mean age ± *SD* = 75.2 ± 2.7 years) were included. At baseline, women underwent *APOE* genotyping and detailed phenotyping for covariates including prevalent falls and fractures, as well as health and lifestyle factors. The association between *APOE ɛ4* and fall-, any fracture-, and hip fracture-related hospitalizations, obtained over 14.5 years from linked health records, was examined using multivariable-adjusted Cox-proportional hazard models. Over 14.5 years, 507 (39.7%) women experienced a fall-related hospitalization and 360 (28.2%) women experienced a fracture-related hospitalization, including 143 (11.2%) attributed to a hip fracture. In multivariable-adjusted models, compared to noncarriers, *APOE ɛ4* carriers (*n* = 297, 23.3%) had greater risk for a fall- (hazard ratio [HR] 1.48, 95% CI: 1.22–1.81), fracture- (HR 1.28, 95% CI: 1.01–1.63), or hip fracture-related hospitalization (HR 1.83, 95% CI: 1.29–2.61). The estimates remained similar when specific fall and fracture risk factors (fear of falling, plasma 25-hydroxyvitamin D, grip strength, timed up-and-go, hip BMD, vitamin K status, prevalent diabetes, HbA1c, cholesterol, and abbreviated mental test score) were added to the multivariable model. In conclusion, *APOE ɛ4* is a potential risk factor for fall- and fracture-related hospitalization in community-dwelling older women. Screening for *APOE ɛ4* could provide clinicians an opportunity to direct higher-risk individuals to appropriate intervention strategies.

Age-related declines in musculoskeletal health, often considered major risk factors for falls and fractures, are a major public health concern for older populations. Specifically, falls are experienced in about 30% of adults older than 65 years (1). In this age group, falls are the leading cause of injury-related hospitalizations (eg, hip fracture), often resulting in decreased independence and quality of life (2). This is exacerbated by osteoporosis with approximately 1 in 2 women and 1 in 5 men aged >50 years expected to experience an osteoporosis-related fracture (3). Of these, hip fractures are the most clinically relevant as they are strongly linked with increased incidence of morbidity and mortality (4). The burden of falls and fractures is anticipated to increase considerably in coming decades in parallel with societal aging, underscoring the need to identify novel risk factors that may improve the efficacy of current screening practices.

Older women are at a higher risk of falling than men (5,6), likely due to both age-related declines in muscle strength and physical function (7,8) and the dramatic deterioration of the structural integrity of bone following menopause (9). The simultaneous presence of impaired muscle and bone health leads older women to have a particular predisposition to sustaining fall-related fractures (5,6). Consequently, increasing attention has been given to the pursuit of biomarkers that may help identify those at high risk of falls and fractures, and ultimately enhance preventative and therapeutic strategies.

Genetic studies indicate that several aspects of bone health, such as bone turnover and bone mineral density (BMD) are highly heritable (10,11). Although recent data suggest that fall risk may also have a genetic component (12), the role of genetics in falls risk remains largely unclear. Therefore, examining the impact of genetic variation on fall and fracture outcomes is a logical avenue for biomarker research. Although a myriad of genes likely contributes to the overall heritability of these phenotypes, one that appears to be particularly promising is the apolipoprotein E (*APOE*) gene. *APOE* has 3 principal alleles, *ɛ2* (*APOE ɛ2*), *ɛ3* (*APOE ɛ3*), and *ɛ4* (*APOE ɛ4*), with the latter being most renowned for its robust association with the risk of dementia, including Alzheimer’s disease (13,14). Notably, even preclinical Alzheimer’s disease is linked with higher falls risk (15). Interestingly, *APOE ɛ4* may be a risk factor for poor bone health through its association with dysregulated lipid homeostasis (16), and potentially reduced vitamin K availability (17), an essential nutrient linked to falls and fracture (18,19). Evidence is conflicting, however, as *APOE ɛ4* has been associated with increased fracture risk and/or poorer BMD (20–22), whereas others have reported no association (23,24). Moreover, despite the role of *APOE ɛ4* in cognition (14), and the nexus between cognition and physical function (25), the relationship between *APOE ɛ4* carrier status and fall risk remains unknown. There are also data indicating the *APOE ɛ4* allele is associated with a more rapid decline in metrics of gait variability (26), which may have further consequences for fall risk.

Given approximately 1 in 4 older women carry the *APOE ɛ4* allele (27), establishing whether its presence is related to fall- and/or fracture-related hospitalization risk may help uncover a scalable screening strategy for identifying older adults at risk of poor musculoskeletal outcomes. Moreover, *APOE ɛ4* genotyping can be performed at any stage of adulthood, and could therefore prompt timely preventative strategies. Herein, we examined if the presence of the *APOE ɛ4* allele increased the long-term risk for fall- and fracture-related hospitalizations in a well-characterized cohort of community-dwelling older women.

## Method

### Study Population

The study population originated from the Perth Longitudinal Study of Ageing Women (PLSAW), which includes 1 500 community-dwelling women aged 70 years or older recruited using the electoral roll. PLSAW is composed of an initial 5-year, double-blind, randomized controlled trial investigating calcium supplementation for fracture prevention (28), followed by 10 additional years of clinic visits and observation. As PLSAW was completed before the clinical trials registry, it was registered retrospectively in the Australian New Zealand Clinical Trials Registry (ACTRN12615000750583). Of the initial 1 500 women, 224 were excluded because of vitamin D supplementation (*n* = 40), *APOE* genotyping not being available (*n* = 159), and missing covariate or outcome data (*n* = 25; Supplementary Figure 1). A total of 1 276 women were available for analysis. Ethics approval for the initial 5-year trial and the subsequent 10-year follow-up was granted by the Human Research Ethics Committee at the University of Western Australia and the Western Australian Department of Health (ethics number #2009/24). Written informed consent was obtained from all participants, including authorization for future access to Western Australian Department of Health Data.

### Baseline Assessments

Height and weight were measured using a wall-mounted stadiometer and digital scales to determine body mass index (BMI, kg/m^2^). Smoking history and physical activity were assessed via questionnaire, detailed in Supplementary Material. Previous falls were determined by asking participants if they had fallen in the 3 months prior to the baseline clinical visit. Prevalent fractures were determined at baseline by asking participants the age and location of fractures sustained after the age of 50 years. Only fractures due to minimal trauma, defined as falling from a height of 1 m or less, were considered, excluding fractures of the face, skull, fingers, or toes (28). Detailed methodology for how DXA-derived total hip BMD, abbreviated mental test score (AMTS) (29), diabetes prevalence, timed up-and-go (TUG) performance, grip strength, and fear of falling data were collected is included in Supplementary Material. Participants had blood samples collected at their baseline clinic visit after an overnight fast, which were subsequently stored at −80°C. Detailed description of the measurement methods and coefficient of variation for HbA1c, plasma 25-hydroxyvitamin D2 and D3 (expressed as total 25OHD), cholesterol, and osteocalcin are provided in Supplementary Material.

### *APOE* Genotyping

Genotyping for *APOE* in this cohort has been described previously (22). Genomic DNA was extracted and purified from whole blood samples collected at baseline. A 227 bp region of the *APOE* gene, which spans polymorphic sites at codons 112 and 158 results in several cutting sites for the CFo1 restriction endonuclease (30), was amplified by polymerase chain reaction using oligonucleotide primers (31). Restriction digests were electrophoresed on 20% acrylamide gels, resulting in DNA fragments unique for each isotype and coded *APOE ɛ2*, *APOE ɛ3*, and *APOE ɛ4*, as previously described (31).

### Fall- and Fracture-Related Hospitalization

Fall- and fracture-related hospitalization data were obtained from the Western Australia Hospital Morbidity Data Collection (HMDC) using the Western Australian Data Linkage System, providing a complete validated record of every participant’s primary diagnosis at hospital discharge using coded data from all hospitals in Western Australia. The HMDC records of all participants were obtained from their baseline visit (1998) and over the next 14.5 years for fall- and fracture-related hospitalization, allowing for ascertainment independent of patient report with the associated problems such as loss to follow-up. Diagnosis codes were defined using the International Classification of Diseases, Injuries, and Causes of Death: Clinical Modification (ICD‐9‐CM) codes for 1998 to 1999 (32), mapped to the ICD‐10 Australian Modification (ICD‐10‐AM) for 1999 to 2013 (33). Hip and fracture‐related hospitalizations were identified using ICD-10 codes S02, S12, S22, S32, S42, S52, S62, S72, S82, S92, M80, T02, T08, T10, T12, and T14.2, excluding fractures of the face (S02.2–S02.6), fingers (S62.5–S62.7), and toes (S92.4–S92.5), or those caused by motor vehicle injuries (External Cause of Injury codes V00-V99). Fall-related hospitalizations were identified using ICD-10 codes W01, W05, W06, WO7, W08, W10, W18, and W19.

### Statistical Analysis

Kaplan–Meier survival analysis examined the univariate association of *APOE ɛ4* presence with fall and fracture hospitalizations. Cox-proportional hazards regression models were used to investigate the association between *APOE ɛ4* presence and fall and fracture outcomes. Two models were run: (1) minimally adjusted: age, treatment code (placebo/calcium) and BMI; and (2) multivariable-adjusted: minimally adjusted model plus smoking history, physical activity, prevalent fracture, and prevalent falls. No violations of the Cox proportional hazards assumptions were detected. All analyses were performed using IBM SPSS (V29, Armonk, NY).

### Additional Analyses

We undertook additional analyses where total hip BMD, cognitive impairment (AMTS < 8), TUG, grip strength, fear of falling, prevalent diabetes, HbA1c, plasma 25OHD (and the season the sample was collected), total cholesterol (and the date of lipid testing), and ucOC:tOC (bone-related biomarker of vitamin K status) were individually included as additional covariates in the multivariable-adjusted models, due to their suggested link to fall and fracture outcomes (5,6,18,19,34–37).

## Results

Participant baseline characteristics are in Table 1. A total of 297 (23.2%) participants carried the *APOE ɛ4* allele, and no statistically significant differences in baseline characteristics were observed between carriers and noncarriers, apart from the number of women with potential cognitive impairment (11 [3.7%] vs 16 [1.6%], respectively) and total cholesterol levels.

**Table 1.** Baseline Characteristics Stratified by *APOE Ɛ4* Presence

Demographics	All Participants	No *APOE ɛ4*	*APOE ɛ4*
Number	1 276	979	297
Age, y	75.2 ± 2.7	75.2 ± 2.7	75.0 ± 2.7
Body mass index (BMI), kg/m^2^	27.2 ± 4.7	27.2 ± 4.8	27.2 ± 4.4
Randomization			
Placebo, yes (%)	637 (49.9)	498 (50.9)	139 (46.8)
Calcium, yes (%)	639 (50.1)	481 (49.1)	158 (53.2)
Smoker ever, yes (%)	467 (36.6)	359 (36.7)	108 (36.4)
Physical activity, kJ/day	112 (34–202)	110 (29–199)	114 (37–221)
Prevalent fracture from age 50 y, yes (%)	344 (27.0)	272 (27.8)	72 (24.2)
Prevalent falls, yes (%)	152 (11.9)	114 (11.6)	38 (12.8)
Total hip BMD^*^, g/cm^2^	0.814 ± 0.125	0.817 ± 0.127	0.803 ± 0.117
Timed up-and-go performance^†^, s	9.9 ± 3.0	9.9 ± 2.8	10.0 ± 3.6
Grip strength^‡^, kg	20.6 ± 4.6	20.6 ± 4.6	20.3 ± 4.6
Fear of falling^§^, yes (%)	343 (27.0)	271 (27.8)	72 (24.3)
Prevalent diabetes, yes (%)	78 (6.1)	61 (6.2)	17 (5.7)
HbA1c^‖^, %	5.3 ± 0.7	5.3 ± 0.7	5.3 ± 0.8
Plasma 25OHD^¶^			
<50 nmol/L, yes (%)	330 (28.1)	250 (27.8)	80 (29.0)
50–<75 nmol/L, yes (%)	433 (36.9)	328 (36.5)	105 (38.0)
≥75 nmol/L, yes (%)	412 (35.1)	321 (35.7)	91 (33.0)
Season vitamin D sample taken^¶^			
Winter/Spring, yes (%)	882 (75.1)	670 (74.5)	212 (76.8)
Summer/Autumn, yes (%)	293 (24.9)	229 (25.5)	64 (23.2)
Total cholesterol^#^, mg/dL	226 ± 42	**224 ± 41**	**230 ± 46**
ucOC:tOC^‖^	0.49 ± 0.12	0.49 ± 0.12	0.49 ± 0.13
Impaired cognitive function (AMTS < 8), yes (%)^**^	27 (2.1)	**16 (1.6)**	**11 (3.7)**

*Notes*: AMTS = abbreviated mental test score; BMD = bone mineral density; HbA1c = glycated hemoglobin; 25OHD = plasma 25-hydroxyvitamin D; ucOC:tOC = ratio of undercarboxylated osteocalcin to total osteocalcin. Data expressed as mean ± S*D*, median (interquartile range), or number and (%). Bolded values represent significant differences (*p* value < .05) between *APOE ɛ4* categories using independent sample *t* test, Chi-square test, or Mann–Whitney *U* test where appropriate.

^*^ *n* = 1 093.

^†^ *n* = 1 274.

^‡^ *n* = 1 265.

^§^ *n* = 1 272.

^‖^ *n* = 1 204.

^¶^ *n* = 1 175.

^#^ *n* = 1 136.

^**^ *n* = 1 275.

### *APOE ɛ4* and Fall- and Fracture-Related Hospitalizations

**Figure 1.**
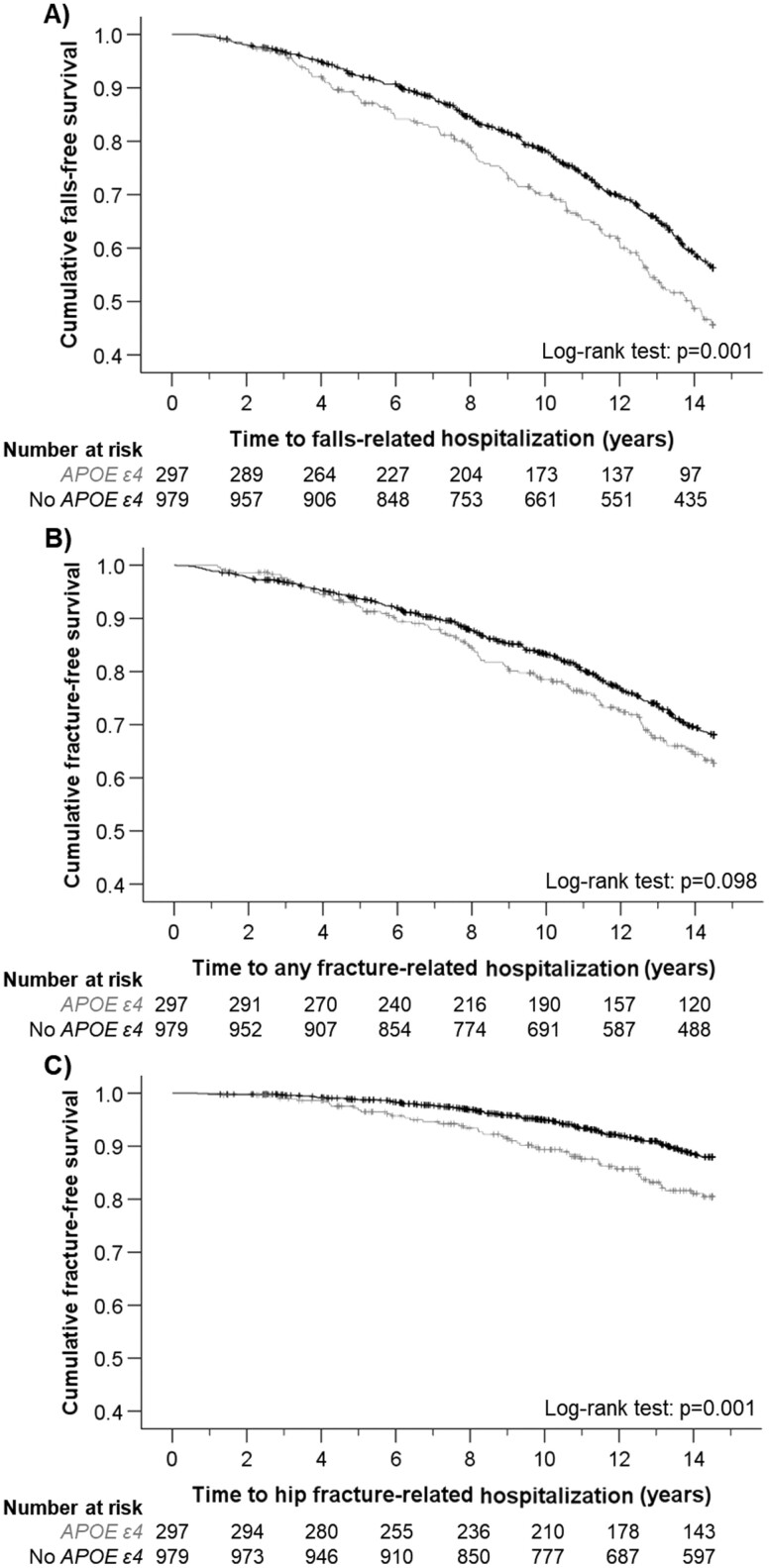
Kaplan–Meier survival curve according to *APOE ɛ4* status for (A) fall-related hospitalization, (B) any fracture-related hospitalization, and (C) hip fracture-related hospitalization. No *APOE ɛ4* and *APOE ɛ4* are represented by black and gray lines, respectively.

Over 14.5 years, the mean ± *SD* patient follow-up period was 11.0 ± 4.0 years for a fall-related hospitalization (14 028 person years), 11.3 ± 4.0 years for any fracture-related hospitalization (14 470 person years), and 12.2 ± 3.4 years for a hip fracture-related hospitalization (15 604 person years). Across the follow-up, 507 (39.7%) women experienced a fall-related hospitalization, 360 (28.2%) women experienced a fracture-related hospitalization, and 143 (11.2%) women experienced a hip fracture-related hospitalization. Kaplan–Meier survival curves indicated that women carrying the *APOE ɛ4* allele had a higher falls risk and hip-fracture risk, compared to noncarriers (Figure 1). In multivariable-adjusted models, *APOE ɛ4* carriers had a 48% greater hazard for a fall-related hospitalization, 28% greater hazard for a fracture-related hospitalization, and 83% greater hazard for a hip fracture-related hospitalization, compared to women without *APOE ɛ4* (Table 2).

**Table 2.** Hazard Ratios for Falls and Fracture Risk by *APOE ɛ4* Presence

	Number of Events (%)	Minimally Adjusted^*^	Multivariable Adjusted^†^
		HR (95% CI)	HR (95% CI)
Fall-related hospitalization			
No *APOE ɛ4*	372/979 (38.0)	**1 (reference)**	**1 (reference)**
* APOE ɛ4*	135/297 (45.5)	**1.45 (1.19–1.77)**	**1.48 (1.22–1.81)**
Any fracture-related hospitalization			
No *APOE ɛ4*	269/979 (27.5)	1 (reference)	**1 (reference)**
* APOE ɛ4*	91/297 (30.6)	1.26 (0.99–1.60)	**1.28 (1.01–1.63)**
Hip fracture-related hospitalization			
No *APOE ɛ4*	97/979 (9.9)	**1 (reference)**	**1 (reference)**
* APOE ɛ4*	46/297 (15.5)	**1.84 (1.29–2.61)**	**1.83 (1.29–2.61)**

*Notes*: *n* = 1 276. Bolded values represent significant differences. Hazard ratios (95% CI) analyzed using Cox-proportional hazard models. BMI = body mass index; HR = hazard ratio.

^*^Minimally adjusted = age, treatment code, and BMI.

^†^Multivariable adjusted = minimally adjusted model plus smoked ever, self-reported prevalent falls, prevalent fractures, and physical activity.

### Additional Analyses

The associations between *APOE ɛ4* presence and falls or fracture outcomes were consistent when impaired cognitive function (AMTS < 8), TUG performance, grip strength, fear of falling, prevalent diabetes, HbA1c, vitamin D status (and season the sample was collected), total cholesterol (and the date of lipid testing), and ucOC:tOC as a biomarker of vitamin K status were each added to the multivariable-adjusted models (Table 3), with the exception that the risk of any fracture hospitalization was slightly attenuated and no longer significant when hip BMD, TUG performance, total cholesterol, or ucOC:tOC were included.

**Table 3.** Hazard Ratios for Falls and Fracture Risk by *APOE ɛ4* Presence

	Fall-Related Hospitalization		Any Fracture-Related Hospitalization		Hip Fracture-Related Hospitalization	
	Number of Events (%)	HR (95% CI)	Number of Events (%)	HR (95% CI)	Number of Events (%)	HR (95% CI)
Multivariable adjusted + total hip BMD^*^						
No *APOE ɛ4*	318/841 (37.8)	**1 (reference)**	233/841 (27.7)	1 (reference)	87/841 (10.3)	**1 (reference)**
* APOE ɛ4*	114/252 (45.2)	**1.44 (1.16–1.79)**	76/252 (30.2)	1.18 (0.91–1.53)	40/252 (15.9)	**1.79 (1.22–2.61)**
Multivariable adjusted + TUG^†^						
No *APOE ɛ4*	372/978 (38.0)	**1 (reference)**	269/978 (27.5)	1 (reference)	97/978 (9.9)	**1 (reference)**
* APOE ɛ4*	134/296 (45.3)	**1.46 (1.20–1.79)**	90/296 (30.4)	1.26 (0.99–1.60)	46/296 (15.5)	**1.85 (1.30–2.63)**
Multivariable adjusted + grip strength^‡^						
No *APOE ɛ4*	370/975 (37.9)	**1 (reference)**	267/975 (27.4)	**1 (reference)**	96/975 (9.8)	**1 (reference)**
* APOE ɛ4*	135/294 (45.9)	**1.46 (1.20–1.78)**	91/294 (31.0)	**1.28 (1.00–1.62)**	46/294 (15.6)	**1.83 (1.29–2.61)**
Multivariable adjusted + fear of falling^§^						
No *APOE ɛ4*	372/976 (38.1)	**1 (reference)**	269/976 (27.6)	**1 (reference)**	97/976 (9.9)	**1 (reference)**
* APOE ɛ4*	135/296 (45.6)	**1.53 (1.25–1.86)**	91/296 (30.7)	**1.30 (1.02–1.65)**	46/296 (15.5)	**1.86 (1.31–2.64)**
Multivariable adjusted + prevalent diabetes^‖^						
No *APOE ɛ4*	372/979 (38.0)	**1 (reference)**	269/979 (27.5)	**1 (reference)**	97/979 (9.9)	**1 (reference)**
* APOE ɛ4*	135/297 (45.5)	**1.50 (1.23–1.83)**	91/297 (30.6)	**1.30 (1.02–1.65)**	46/297 (15.5)	**1.89 (1.32–2.68)**
Multivariable adjusted + HbA1c^¶^						
No *APOE ɛ4*	343/916 (37.4)	**1 (reference)**	248/916 (27.1)	**1 (reference)**	91/916 (9.9)	**1 (reference)**
* APOE ɛ4*	130/288 (45.1)	**1.49 (1.22–1.83)**	89/288 (30.9)	**1.32 (1.03–1.68)**	44/288 (15.3)	**1.85 (1.29–2.66)**
Multivariable adjusted + 25OHD^#^						
No *APOE ɛ4*	344/899 (38.3)	**1 (reference)**	242/899 (26.9)	**1 (reference)**	88/899 (9.8)	**1 (reference)**
* APOE ɛ4*	127/276 (46.0)	**1.49 (1.21–1.82)**	85/276 (30.8)	**1.31 (1.02–1.68)**	43/276 (15.6)	**1.85 (1.28–2.67)**
Multivariable adjusted + total cholesterol^**^						
No *APOE ɛ4*	330/875 (37.7)	**1 (reference)**	237/875 (27.1)	1 (reference)	88/875 (10.1)	**1 (reference)**
* APOE ɛ4*	117/261 (44.8)	**1.50 (1.21–1.86)**	76/261 (29.1)	1.24 (0.95–1.61)	36/261 (13.8)	**1.62 (1.09–2.39)**
Multivariable adjusted + ucOC:tOC^††^						
No *APOE ɛ4*	353/923 (38.2)	**1 (reference)**	254/923 (27.5)	1 (reference)	95/923 (10.3)	**1 (reference)**
* APOE ɛ4*	126/281 (44.8)	**1.43 (1.16–1.75)**	83/281 (29.5)	1.23 (0.96–1.58)	39/281 (13.9)	**1.55 (1.07–2.26)**
Multivariable adjusted + impaired cognitive function^‡‡^						
No *APOE ɛ4*	372/979 (38.0)	**1 (reference)**	269/979 (27.5)	**1 (reference)**	97/979 (9.9)	**1 (reference)**
* APOE ɛ4*	135/296 (45.6)	**1.49 (1.22–1.82)**	91/296 (30.7)	**1.30 (1.02–1.65)**	46/296 (15.5)	**1.85 (1.30–2.63)**

*Notes*: BMD = bone mineral density; HbA1c = glycated hemoglobin; HR = hazard ratio; TUG = timed up-and-go; 25OHD = plasma 25-hydroxyVitamin D; ucOC:tOC = ratio of undercarboxylated osteocalcin to total osteocalcin. Multivariable adjusted = age, treatment code, BMI, smoked ever, self-reported prevalent falls, prevalent fractures, and physical activity. Bolded values represent significant differences. Hazard ratios (95% CI) analyzed using Cox-proportional hazard models.

^*^ *n* = 1 093.

^†^ *n* = 1 274.

^‡^ *n* = 1 269.

^§^ *n* = 1 272.

^‖^ *n* = 1 276.

^¶^ *n* = 1 204.

^#^Multivariable adjusted plus plasma 25OHD and season 25OHD sample taken (*n* = 1 175).

^**^Multivariable adjusted plus total cholesterol and date of lipid testing (n = 1 136).

^††^ *n* = 1 204.

^‡‡^Multivariable adjusted plus impaired cognitive function defined as abbreviated mental test score (AMTS) < 8 (*n* = 1 275).

## Discussion

This study demonstrates an increased long-term risk of fall- and fracture-related hospitalizations in community-dwelling older women carrying the *APOE ɛ4* allele. The novel finding is that the *APOE ɛ4* allele substantially increases the risk of falling. The relevance of *APOE ɛ4* to fall- and fracture-related hospitalizations may be driven by the robust links between *APOE ɛ4*, cognitive impairment (14) and cognitive decline with injurious falls (25).

Although there is a paucity of data relating to *APOE ɛ4* and falls, the *APOE ɛ4* allele is a reported risk factor for the development of gait impairment, a likely contributor (26). However, the association between *APOE ɛ4* and fall-related hospitalization risk in the present study withstood adjustment for baseline TUG performance, suggesting the underlying mechanism to be somewhat independent of physical function. Nevertheless, previous data suggest that general measures of gait, such as gait speed do not differ according to *APOE ɛ4* status, whereas more specific measures of gait, such as stride length and stride time variability, which may be particularly relevant to fall risk, do differ (26,38). Therefore, although TUG performance did not seem to affect the relationship between *APOE ɛ4* and fall-related hospitalization risk in the present study, future research incorporating sensitive measures of gait is needed to contextualize these findings.

Interestingly, decline in cognitive function, rather than physical function, has been reported to have greater prognostic accuracy for injurious falls over long follow-ups (35). Possible mechanisms by which poorer cognition may increase falls risk include poor executive function reducing dual-tasking ability and response inhibition, and delayed reaction speed and poorer attention reducing the ability to react to balance perturbations (39,40). Although the proportion of women in the current study who presented with impaired cognitive function (29) at baseline was slightly higher in APOE ɛ4 carriers (*n* = 11, 3.7%) versus noncarriers (*n* = 16, 1.6%), the prevalence was low overall (*n* = 27, 2.1%). This is likely a reflection of women being recruited only if they had a projected survival beyond the 5-year clinical trial. Furthermore, adjustment for AMTS-defined impaired cognition function or the exclusion of these 27 women (data not shown) in our primary analysis did not change the interpretation of our results. In this regard, the low prevalence of impaired cognitive function in our cohort may have precluded an interaction being observed between cognition and fall-related hospitalization risk. Notably, the AMTS alone may not be the optimal tool to screen for cognitive impairment, as some data suggest it may be less sensitive to detect poor cognition, when compared to other screening tools such as the Montreal Cognitive Assessment (41). The AMTS is also a general measure of cognitive function but does not assess individual components of cognition, some of which may be particularly important in predicting falls risk. As such, although in this instance cognitive status did not appear to be a risk factor, future studies incorporating cohorts with diverse ranges in cognitive health and a more comprehensive battery of cognitive assessments to adequately and sensitively assess multiple domains of cognition are needed to contextualize our findings and further elucidate the potential role of cognition in the relationship between *APOE ɛ4* and falls.

In our study, we identified a robust relationship between *APOE ɛ4* status and hip fracture-related hospitalization, with carriers having an 89% greater risk compared to noncarriers. These associations remained unchanged after adjustment for other well-established fall and fracture risk factors, including hip BMD, circulating 25OHD levels, fear of falling, and muscle function measures. Our findings complement previous reports demonstrating *APOE ɛ4* to be a risk factor for fractures (20,21,42), and for the first time, the relevance of *APOE ɛ4* to injurious falls risk. Notably, 23.2% of women in our study carried the *APOE ɛ4* allele, which is similar to other general population estimates of ~25% (43). The high prevalence and concomitant risk of the *APOE ɛ4* allele support its potential use as a screening tool, with relevance beyond cognitive impairment for which it is most renowned for. Better understanding of the relationship between *APOE ɛ4*, fall- and fracture-risk may help guide a targeted delivery of strategies to improve musculoskeletal health and reduce the prevalence of injurious falls and fractures among older adults. In this regard, *APOE ɛ4* screening could help identify older adults at risk of falls and fractures who may benefit from inclusion into therapeutic programs, especially those involving lifestyle intervention (eg, exercise, diet). This approach in early stages of adulthood could assist in supporting musculoskeletal health and minimize declines over time.

The association between *APOE ɛ4* and bone health is particularly robust in women, with studies having shown associations of *APOE ɛ4* with BMD and/or fracture risk (20–22,44,45). While total hip BMD did not differ by *APOE ɛ4* status in the unadjusted analyses presented here, published data from the current cohort shows that women with *APOE ɛ4* had lower hip BMD and calcaneal ultrasound parameters when adjusted for age, BMI, alcohol consumption, and cigarette smoking, compared to women without *APOE ɛ4* (22). In that analysis from this cohort, no difference in risk of prevalent or incident clinical fracture was reported over 2 years between women with and without *APOE ɛ4* (22). This contrasting result may be explained by the longer follow-up in the current analysis, where separation in the Kaplan–Meier curves for falls and fractures was most apparent from approximately 5 years of follow-up. In contrast, the associations shown between *APOE ɛ4* and bone health in men have been weaker, or absent (24,45,46). Nevertheless, some cross-sectional studies have not shown associations between *APOE ɛ4* and bone outcomes in females, although small sample size (*n* = 147) (47) or low *APOE ɛ4* prevalence (7%) (48) may have influenced results. Notwithstanding such studies, research broadly favors a deleterious effect of *APOE ɛ4*, particularly in women. Our study adds substantive support, providing evidence over a long follow-up from a well-characterized cohort with verified outcome records and comprehensive adjustment for known risk factors. Future prospective studies are needed, however, to confirm the extent to which sex may mediate the relationship between *APOE ɛ4*, bone, and functional outcomes.

There are several putative pathways that may underpin the associations between *APOE ɛ4* status and fractures. First, *APOE ɛ4* has been linked with dysregulated lipid metabolism and transport, elevated cholesterol, and atherosclerosis risk (16,49), all of which can negatively affect bone health (34,50). Second, there is evidence, although conflicting, that *APOE* may affect bone properties through its involvement in transporting vitamin K, an important nutrient involved in the carboxylation of osteocalcin and other bone-related proteins (51). In our study, total cholesterol was higher among women with *APOE ɛ4* compared to those without, although we showed no difference in the ucOC:tOC ratio, suggesting no difference in vitamin K status. Although the hazard ratios for any fracture were attenuated when either total cholesterol or ucOC:tOC were included in the multivariable models, the associations with hip fracture- and fall-related hospitalizations remained robust and consistent. Thus, the evidence of interactions between *APOE* genotypes, lipid metabolism, and/or vitamin K is conflicting (52,53). Future studies are needed to illuminate the pertinence of their relationship to bone health.

Study strengths include the prospective, population-based study design, the long-term follow-up (14.5 years), and verified fall- and fracture‐related hospitalizations being obtained from linked health records, independent of self‐report. Additionally, our analyses were adjusted for a panel of relevant covariates that have largely been unaccounted for in previous studies. There are also several limitations. This was an observational study so causality cannot be established. Considering only older women were included in the study, results should not be generalized to other populations. Nonetheless, older women are arguably the most relevant target population for this work, having the highest predisposition to injurious falls and fractures (5,6), and the proportion of women carrying the *APOE ɛ4* allele in our study was comparable to other Australian estimates (27). We also cannot rule out the possibility of residual confounding on these results, hence further research is required to explore the potential mechanisms underpinning the relationship between *APOE ɛ4*, falls, and fractures. Finally, we only included falls and fractures that resulted in hospitalization. Such events have considerable healthcare burden, especially with an aging population, meaning that our data provides an opportunity to examine the most serious falls and fractures that are less frequently reported.

In conclusion, our findings suggest that *APOE ɛ4* status has value for identifying fall- and fracture-related hospitalization risk in older women. Moreover, considering *APOE ɛ4* carriers are also at risk of cognitive impairment, the implementation of primary prevention strategies, including exercise, may be particularly beneficial for this cohort. Although *APOE ɛ4* screening may help guide the deployment of interventions seeking to prevent falls and fractures, many of the corresponding risk factors, such as BMD and muscle strength, are heritable traits that are mediated by a network of genes. Accordingly, the proficiency of genotypic screening would likely be strengthened by including a panel of risk polymorphisms. Further research is therefore needed to (a) identify other prevalent polymorphisms that predispose individuals to falls and fractures and (b) determine the cumulative prognostic power of these polymorphisms, including *APOE ɛ4*, for fall and fracture risk.

## Supplementary Material

glae134_suppl_Supplementary_Materials
