# Supplementary material for: Apolipoprotein ɛ4 Is Associated With Increased Risk of Fall- and Fracture-Related Hospitalization: The Perth Longitudinal Study of Ageing Women
Source: J Gerontol A Biol Sci Med Sci. 2024 May 20;79(8):glae134. doi: 10.1093/gerona/glae134 (PMC11212482; doi:10.1093/gerona/glae134)
Supplement: glae134_suppl_Supplementary_Materials [file glae134_suppl_supplementary_materials.docx]

**Supplementary Material**

Excluded (n = 184)

- Missing *APOE ɛ4* genotype (n=159)
- Missing BMI (n=2)
- Missing prevalent falls or fracture (n=21)
- Missing smoking status (n=2)

14.5 year fall and fracture-related hospitalisations retrieved from the Western Australia Hospital Morbidity Data System for all participating women who remained in Western Australia

Assessed for eligibility

(n=1460)

Eligible for analysis

(n=1276)

**eFigure 1. Participant flow diagram**

**Supplementary Text**

**Questionnaires**

Participants classified as either a non-smoker or ever-smoker, which included current smokers and those who smoked at least one cigarette per day for three or more months at any time in their life. For physical activity, women completed a questionnaire that included the following questions: “Do you participate in any sports recreation or regular physical exercise?”, “Please list any sports recreation or regular physical activity, including walking, that you undertook in the last three months.” Those who answered “yes” to the first question were asked to list up to four activities and the duration (in hours/week) that they had engaged in each activity. Women who answered no to the activity question were classified as being sedentary. Activity levels in the active women were calculated in kcal/day for each individual based on body weight and established energy costs of listed activities (1).

**Bone mineral density**

Total hip BMD was measured by dual-energy X-ray absorptiometry (DXA; Hologic Acclaim 4500A, Hologic Corp, Waltham, MA, USA). The coefficient of variation (CV) at the total hip was 1.2% in our laboratory (2).

**Muscle function**

The timed up-and-go test (TUG), assessed as the time taken to rise from a chair, walk three meters, turn, and return to sit on the chair, was used as a measure of physical function. Grip strength, assessed as the peak value recorded using an isometric Jamar hand dynamometer, was used as a measure of muscle strength. Inter-observer CVs were 7% and 6% for grip strength and TUG, respectively, in our laboratory based on a random sample of 30 women.

**Prevalent diabetes**

Prevalent diabetes was determined by insulin or oral hypoglycaemic medication use, confirmed where possible by primary care physicians and classified according to the International Classification of Primary Care-Plus system (T89001-T90009). The coding methodology allows aggregation of different terms for similar pathologic entities as defined by the ICD-10 coding system.

**Biomarkers**

HbA1c was measured using standard colorimetric and enzymatic methods using a Cobas Integra 800 analyser with reagents supplied by Roche Diagnostics (Castle Hill, NSW, Australia). Plasma 25-hydroxyvitamin D2 (25OHD2) and D3 (25OHD3) were measured using a validated liquid chromatography with tandem mass spectrometry (LC-MS/MS) method adopted at the RDDT Laboratories (Bundoora, VIC, Australia) in 1,175 women (3).

Total plasma 25OHD was calculated by summing 25OHD2 and 25OHD3. CVs were 10.1% at a 25OHD2 mean concentration of 12 nmol/L and 11.3% at a 25OHD3 mean concentration of 60 nmol/L. The season when the blood sample was recorded and categorised as summer/autumn and winter/spring.

Serum lipid profiles, including total cholesterol, were measured using a Hitachi 917 auto analyser (Roche Diagnostics, Mannheim, Germany). A subset (n=561) of these samples were re-analysed in 2019 using the Abbott ARCHITECT ci16200 Integrated System (Abbott, Abbott Park, IL, USA). We preferentially used the 2019 cholesterol values in our analysis when available and included the date of lipid testing in models that included cholesterol to account for differences in time or assay.

Serum total osteocalcin (tOC) was measured by sandwich electrochemiluminescence immunoassay using the Roche Cobas N-Mid Osteocalcin assay (Roche Diagnostics, Mannheim, Germany). CVs were 2.3% at 18 ng/mL and 4.8% at 90 ng/mL. Serum undercarboxylated osteocalcin (ucOC) was measured by the same reagent assay with pre-treatment of the serum samples using 5 mg/mL of hydroxyapatite (Calbiochem, MilliporeSigma, Burlington, MA, USA) (4,5). CVs for percentage binding of carboxylated OC were 12% at 15 ng/mL and 8% at 100 ng/mL. ucOC was divided by tOC to determine the ratio of undercarboxylated to total osteocalcin (ucOC:tOC). Two participants with implausible ucOC:tOC ratio (i.e., >1.0) were excluded from analyses using ucOC:tOC.

**Supplementary references**

1. Bruce DG, Devine A, Prince RL. Recreational physical activity levels in healthy older women: the importance of fear of falling. J Am Geriatr Soc 2002;50:84-89.

2. Lewis JR, Eggermont CJ, Schousboe JT et al. Association Between Abdominal Aortic Calcification, Bone Mineral Density, and Fracture in Older Women. J Bone Miner Res 2019;34:2052-2060.

3. Maunsell Z, Wright DJ, Rainbow SJ. Routine isotope-dilution liquid chromatography-tandem mass spectrometry assay for simultaneous measurement of the 25-hydroxy metabolites of vitamins D2 and D3. Clin Chem 2005;51:1683-90.

4. Chubb SP, Byrnes E, Manning L et al. Reference intervals for bone turnover markers and their association with incident hip fractures in older men: the Health in Men study. J Clin Endocrinol Metab 2015;100:90-99.

5. Gundberg CM, Nieman SD, Abrams S, Rosen H. Vitamin K status and bone health: an analysis of methods for determination of undercarboxylated osteocalcin. J Clin Endocrinol Metab 1998;83:3258-3266.
